# Predictors and prognostic impact of left ventricular ejection fraction trajectories in patients with ST-segment elevation myocardial infarction

**DOI:** 10.1007/s40520-022-02087-y

**Published:** 2022-02-11

**Authors:** Zhijun Lei, Bingyu Li, Bo Li, Wenhui Peng

**Affiliations:** Department of Cardiology, School of Medicine, Shanghai Tenth People’s Hospital, Tongji University, 301 Middle Yanchang Road, Shanghai, 200072 China

**Keywords:** STEMI, LVEF, Trajectory, Predictors, Prognosis

## Abstract

**Background:**

There is little evidence on left ventricular ejection fraction (LVEF) trajectories after ST-segment elevation myocardial infarction (STEMI).

**Aim:**

We aim to identify the LVEF trajectories after STEMI and explore their predictors and association with prognosis.

**Methods:**

This is a retrospective, observational study of STEMI patients. The LVEF trajectories were identified by the latent class trajectory model in patients with baseline LVEF < 50%. We used logistic regression analysis to investigate the predictors for LVEF trajectories. The Cox proportional hazard model was used to assess the impact of LVEF trajectories on prognosis. The primary outcomes were cardiovascular mortality and heart failure (HF) rehospitalization.

**Results:**

572 of 1179 patients presented with baseline normal LVEF (≥ 50%) and 607 with baseline reduced LVEF (< 50%). Two distinct LVEF trajectories were identified in patients with baseline reduced LVEF: recovered LVEF group and persistently reduced LVEF group. Higher baseline LVEF, lower peak troponin T, non-anterior MI, and lower heart rates were all found to be independently associated with LVEF recovery. After multivariate adjustments, patients with persistently reduced LVEF experienced an increased risk of cardiovascular mortality (HR 7.49, 95% CI 1.94–28.87, *P* = 0.003) and HF rehospitalization (HR 3.54, 95% CI 1.56–8.06 *P* = 0.003) compared to patients with baseline normal LVEF. Patients with recovered LVEF, on the other hand, showed no significant risk of cardiovascular mortality and HF rehospitalization.

**Conclusion:**

Our study indicated two distinct LVEF trajectories after STEMI and that the persistently reduced LVEF trajectory was related to poor prognosis. In addition, several baseline characteristics can predict LVEF recovery.

**Supplementary Information:**

The online version contains supplementary material available at 10.1007/s40520-022-02087-y.

## Introduction

Atherosclerotic cardiovascular disease (ASCVD) remains the leading cause of mortality and morbidity worldwide with an aging population. And ST-segment elevation myocardial infarction (STEMI) is the most severe type [1, 2]. Despite rapid advances in pharmacological and interventional treatment strategies, STEMI remains the leading cause of mortality and heart failure (HF) [1]. Previous data showed that decreased left ventricular ejection fraction (LVEF) is usually recognized in the acute phase of STEMI, and STEMI patients with decreased LVEF are at an increased risk of death [3, 4] and HF [5–7]. Nearly half of STEMI patients with baseline decreased LVEF can improve their LVEF within the first months [8, 9]. The recovery of stunned myocardium following acute occlusions determines the degree of LVEF recovery in the early phase (days–weeks) [10]. In contrast, the long-term improvement in LVEF is related to the left ventricular (LV) remodeling triggered by necrosis, hypertrophy, inflammation, and fibrosis in the infarct zone [11, 12]. Although the current clinical guidelines have recommended repeating echocardiography within 6–12 weeks after discharge to evaluate the need for implantable cardioverter-defibrillator (ICD) [13, 14], studies have shown that the LVEF recovery following acute myocardial infarction (MI) is associated with a reduced risk of cardiac arrest, all-cause mortality, cardiovascular mortality and HF rehospitalization [15–18]. However, current studies of LVEF recovery after MI have only reassessed LVEF one time, with intervals ranging from weeks to a year [15, 17], ignoring the dynamic trajectories of LVEF after STEMI in the reperfusion era. So far, there is a lack of study on the LVEF trajectories in STEMI patients undergoing primary percutaneous coronary intervention (PCI).

Given this knowledge gap, this study aimed to identify the LVEF trajectories over a 3-year follow-up among STEMI patients undergoing primary PCI and explore their predictors and association with long-term cardiovascular mortality and HF rehospitalization.

## Methods

### Study population

**Fig. 1:**
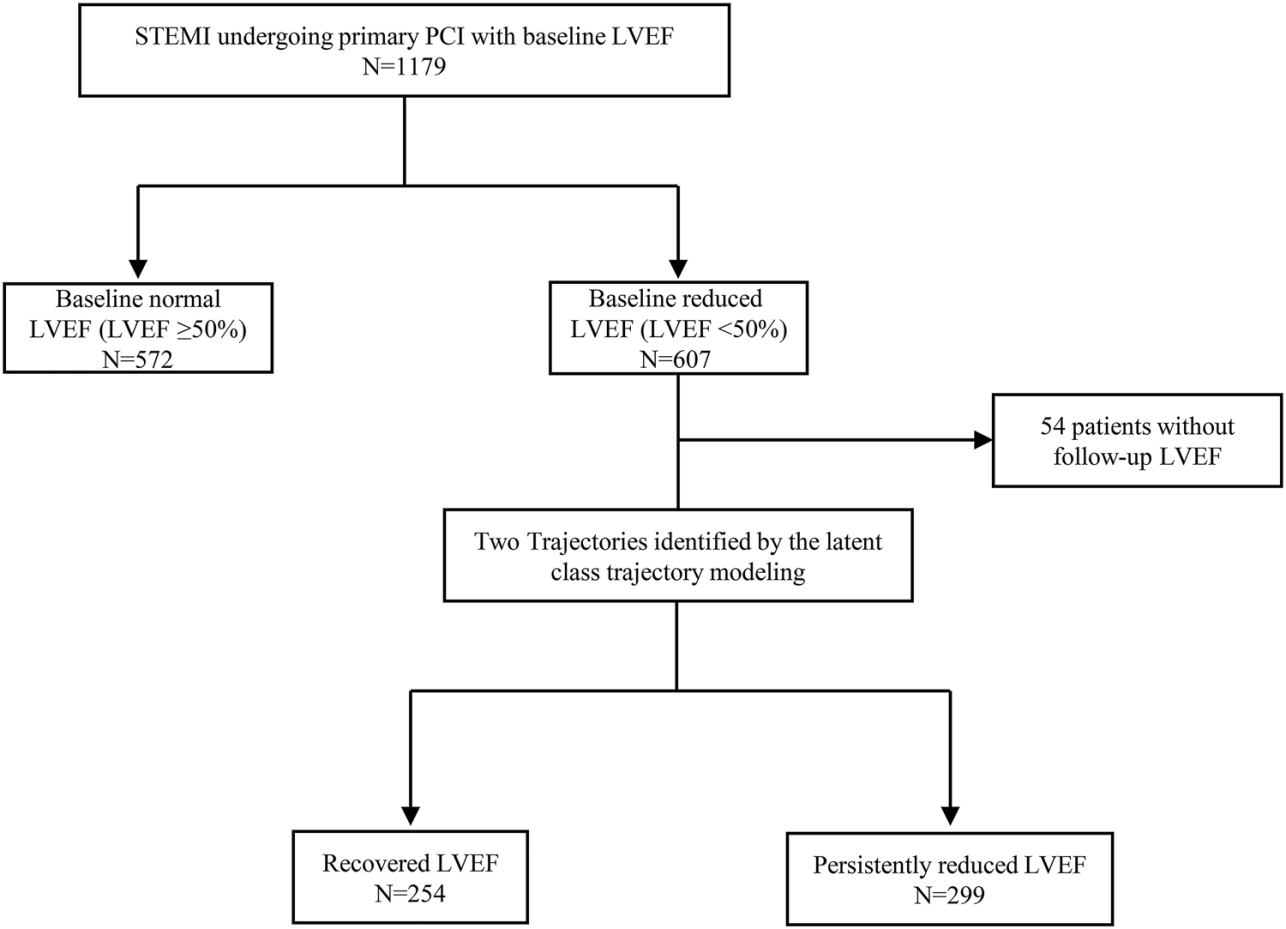
The study flowchart of patients with STEMI undergoing primary PCI. *STEMI* ST-segment elevation myocardial infarction, *PCI* Percutaneous coronary intervention, *LVEF* Left ventricular ejection fraction

A retrospective, observational study was performed for all patients admitted between January 2015 and April 2019 with a diagnosis of STEMI in Shanghai Tenth People’s Hospital. Both baseline and follow-up LVEF were measured by modified Simpson on echocardiography. The baseline LVEF value was measured within 72 h after admission. The follow-up LVEF values were obtained at 1 month, 3 months, 6 months, 9 months, 12 months, 18 months, 24 months, 30 months, and 36 months after discharge. The main inclusion criteria were having had ≥ 2 LVEF values, one at baseline and the other during the follow-up. Exclusion criteria were patients who died in the hospital, without undergoing primary PCI, with previous MI, or without available baseline LVEF value. As shown in the flowchart of patients (Fig. 1), 572 of 1179 eligible patients presented with baseline normal LVEF (≥ 50%) and 607 with baseline reduced LVEF (< 50%). The final cohort consisted of 553 individuals after excluding 54 patients from the latter group who did not follow-up LVEF values. Data on demographics, cardiovascular risk factors, angiographic information, laboratory test, and discharge medications were collected. This study was approved by the Shanghai Tenth People’s Hospital’s Ethics Committee and was carried out following the Declaration of Helsinki.

### Left ventricular ejection fraction assessment

Patients were categorized according to the baseline LVEF: baseline normal LVEF (≥ 50%) and baseline reduced LVEF (< 50%). Patients with a baseline reduced LVEF were further divided into recovered LVEF group and persistently reduced LVEF group according to the latent class trajectory model (LCTM). In summary, patients were stratified into three groups: baseline normal LVEF group (LVEF ≥ 50% at baseline), recovered LVEF group (LVEF < 50% at baseline and recovered over time) and persistently reduced LVEF group (LVEF < 50% at baseline and without recovered over time).

### Study endpoints

The primary endpoints were cardiovascular mortality and HF rehospitalization. Cardiovascular mortality was defined as death attributable to myocardial ischemia and infarction, heart failure, cardiac arrest because of other or unknown cause, or cerebrovascular accident. HF rehospitalization was defined as readmission for HF symptoms requiring an intravenous injecting diuretics, inotropes, vasodilators, or recombinant human brain natriuretic peptide.

### Statistical analysis

According to different types of distribution, continuous variables were displayed as mean ± standard deviation (SD) or median [25th–75th]. Categorical variables were displayed as frequencies and percentages. As appropriate, comparisons for continuous variables were performed by one-way ANOVA, Mann–Whitney *U* test or Kruskal–Wallis test, while comparisons for categorical variables were performed by chi-square or Fisher exact tests.

We used LCTM to differentiate trajectories of LVEF over time in patients with a baseline reduced LVEF. The LCTM can identify latent classes of individuals following similar trends of a determinant over time [19]. Our models adopt second-order polynomials. We calculated the posterior probabilities for each trajectory for every patient to determine the goodness of fit and the proportion of individuals categorized into each class with a posterior probability greater than 0.7, indicating the proportion of individuals categorized in each latent class. We selected the best-fitting number of trajectories among the models with two to five based on a minimum Bayesian Information Criterion (BIC) and a maximum posterior probability [20]. As shown in Supplementary Table 1, two trajectories were selected as the optimum model owing to minimum BIC value and maximum mean posterior probability. The locally weighted error sum of squares (Loess) curves was plotted for each trajectory to show the trend of LVEF over time [21]. We assigned labels to the trajectories (recovered LVEF and persistently reduced LVEF) based on their patterns over time to facilitate interpretability.

We included variates in the univariate logistic regression analysis to evaluate the factors associated with LVEF recovery in patients with a baseline reduced LVEF. Then variates with *P* < 0.05 were entered in a multivariate logistic regression model to identify independent factors of LVEF recovery. Event-free survival curves were plotted by the Kaplan–Meier method and the log-rank test was used for comparisons. The multivariate Cox proportional hazards model was used to calculate hazard ratio (HR) and 95% confidence interval (CI).

Despite the model with two latent classes was the optimum one, we still performed a sensitivity analysis using the model with 3 latent classes to further elucidate our findings owning to its lower BIC and higher mean posterior probability (Supplementary Table 1). We assigned labels to the trajectories (greatly recovered LVEF, mildly recovered LVEF, and persistently reduced LVEF) according to their trend over time.

All tests were two-sided and *P* value < 0.05 was considered significant. All statistical analyses were completed by R software (Version 4.0.5). The LCTM was conducted by packages “lcmm” and “nlme”. The Loess curves were plotted by package “ggplot2”. The survival curves were plotted and analyzed by packages “survival” and “survminer”.

## Results

### LVEF trajectories

**Fig. 2:**
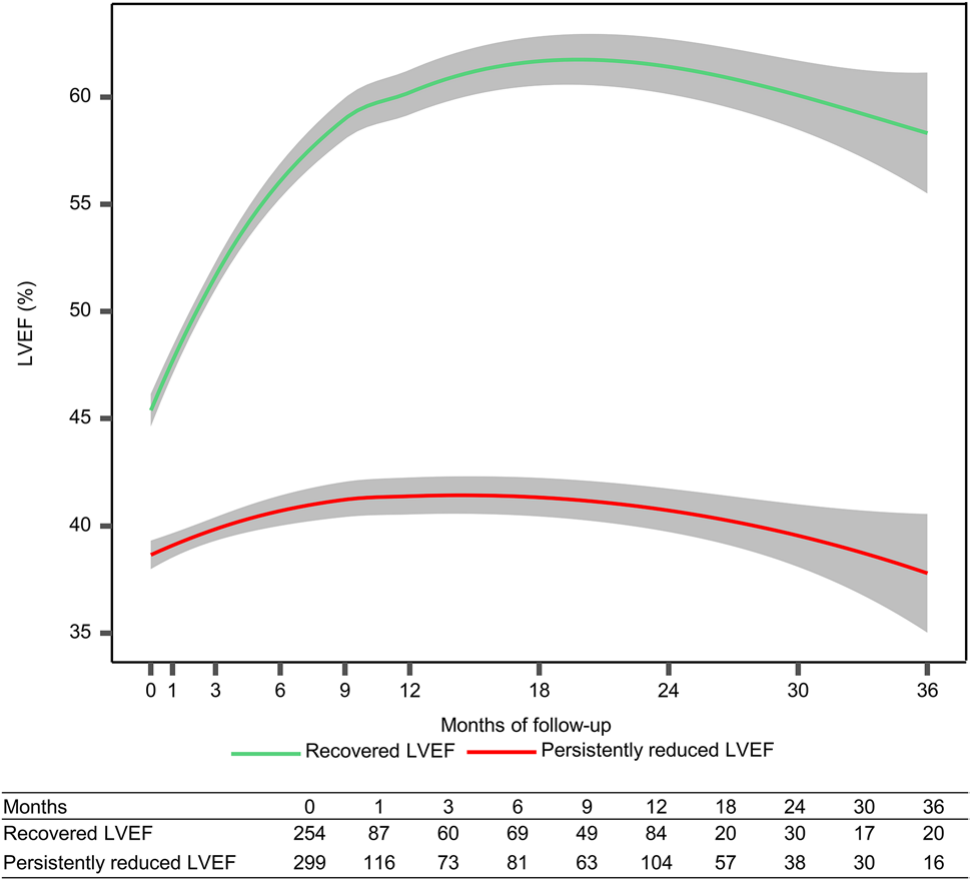
Comparison of long-term LVEF trajectories between patients with recovered LVEF (green) and persistently reduced LVEF (red). *P* = 0.057 for LVEF trajectory changes for patients with persistently reduced LVEF, *P* < 0.001 for LVEF trajectory changes for patients with recovered LVEF; *P* < 0.001 for comparison between the two groups. Shaded regions represent 95% confidence interval. The table shows the number of LVEF values at each time points

A total of 553 patients were included in the final cohort, and 1567 LVEF values were measured. The mean LVEF measurements per patient was 2.8 ± 1.1 and the distribution of the number of LVEF measurements performed per patient was showed in Supplementary Fig. 1. As illustrated in the Loess curves (Fig. 2), 553 patients with a baseline reduced LVEF were assigned into two different LVEF trajectories over a 3-year follow-up based upon the LCTM. They were named the recovered LVEF group (45.9%) and the persistently reduced LVEF group (54.1%) based on their trend over time. In patients with recovered LVEF, the LVEF value increased gradually throughout the first 9 months and reached the plateau (*P* for trajectory < 0.001). In contrast, the LVEF value remained nearly unchanged at a lower level in patients with persistently reduced LVEF (*P* for trajectory = 0.057).

### Baseline characteristics

The baseline characteristics of patients among the 3 groups are shown in Table 1. The baseline LVEF was 57.0% (53.0–60.0%) in patients with baseline normal LVEF, 43.0% (40.0–48.0%) in patients with recovered LVEF and 38.0% (34.0–43.0%) in patients with persistently reduced LVEF (*P* < 0.001). Compared to patients with recovered LVEF, patients with persistently reduced LVEF were older, more likely to have diabetes mellitus, more presented with anterior MI, higher Killip class, and higher heart rates. Angiographic characteristics indicated that the worse TIMI flow before PCI and the usage of intra-aortic balloon pump (IABP) was more common in patients presenting with persistently reduced LVEF. Compared with patients with recovered LVEF, initial creatinine and peak troponin T were lower in patients with baseline normal LVEF and higher in patients with persistently reduced LVEF. Considering the treatment at discharge, patients with persistently reduced LVEF received a significantly higher proportion of β-receptor blockers, but with no difference in the other medications. Supplementary Table 2 displayed that no difference in baseline characteristics was observed in baseline reduced LVEF patients with and without follow-up LVEF.

**Table 1:** Demographic, clinical, and therapeutic characteristics of patients

	Baseline normal LVEF group (*N* = 572)	Recovered LVEF group (*N* = 254)	Persistently reduced LVEF group (*N* = 299)	*P* value
Age, years	61.7 ± 11.3	63.6 ± 11.5	64.8 ± 11.2	< 0.001
Male	478 (83.6%)	206 (81.1%)	252 (84.3%)	0.576
BMI, kg/m^2^	24.5 (22.6–26.6)	24.3 (22.8–26.1)	24.4 (22.8–26.6)	0.895
Hypertension	343 (60.0%)	158 (62.2%)	187 (62.5%)	0.704
Diabetes	181 (31.6%)	90 (35.4%)	123 (41.1%)	0.020
Chronic kidney disease	37 (6.5%)	9 (3.5%)	18 (6.0%)	0.236
Prior stroke	45 (7.9%)	17 (6.7%)	24 (8.0%)	0.807
Prior PCI	29 (5.1%)	6 (2.4%)	8 (2.7%)	0.084
Smoking	358 (62.6%)	154 (60.6%)	180 (60.2%)	0.748
Anterior MI	192 (33.6%)	142 (55.9%)	232 (77.6%)	< 0.001
Killip class II–IV	57 (10.0%)	34 (13.4%)	53 (17.7%)	0.005
SBP, mmHg	133.9 ± 23.2	136.3 ± 24.5	137.2 ± 26.2	0.132
Heart rate, beats/min	75.7 ± 15.1	79.7 ± 20.0	85.8 ± 18.7	< 0.001
Out-of-hospital cardiac arrest	16 (2.8%)	12 (4.7%)	7 (2.3%)	0.227
Multivessel disease	311 (54.4%)	153 (60.2%)	172 (57.7%)	0.262
TIMI flow 0–1 before PCI	330 (57.7%)	168 (66.1%)	217 (72.6%)	< 0.001
Usage of IABP	7 (1.2%)	20 (7.9%)	33 (11.0%)	< 0.001
Baseline LVEF, %	57.0 (53.0–60.0)	43.0 (40.0–48.0)	38.0 (34.0–43.0)	< 0.001
Initial creatinine, umol/L	77.2 (66.7–89.2)	78.4 (65.9–90.2)	79.8 (67.8–97.1)	0.028
Peak troponin *T*, ng/mL	4.56 (2.04–8.53)	5.76 (3.00–9.93)	10.00 (5.21–10.00)	< 0.001
Medication at discharge				
Aspirin	542 (94.8%)	248 (97.6%)	284 (95.0%)	0.166
Anti-P_2_Y_12_ receptors	553 (96.7%)	251 (98.8%)	295 (98.7%)	0.071
Statins	549 (96.0%)	245 (96.5%)	292 (97.7%)	0.436
ACEIs/ARBs	319 (55.8%)	158 (62.2%)	172 (57.5%)	0.224
β-receptor blockers	403 (70.5%)	201 (79.1%)	259 (86.6%)	< 0.001

*LVEF* left ventricular ejection fraction, *BMI* body mass index, *PCI* percutaneous coronary intervention, *MI* myocardial infarction, *SBP* systolic blood pressure, *TIMI* thrombolysis in myocardial infarction, *IABP* intra-aortic balloon pump, *ACEIs* angiotensin-converting enzyme inhibitors, *ARBs* angiotensin II receptor blockers

### Predictors of LVEF recovery

The predictors of LVEF recovery in patients with a baseline reduced LVEF are displayed in Table 2. In univariate analysis, factors including anterior MI, heart rates, baseline LVEF, peak troponin T, and prescription with β-receptor blockers were associated with LVEF recovery in patients with a baseline reduced LVEF. After using multivariate logistic regression analysis, anterior MI (OR 0.55, 95% CI 0.36–0.84, *P* = 0.006), heart rates (OR 0.88 per 10 bpm increase, 95% CI 0.79–0.98, *P* = 0.021), baseline LVEF (OR 1.71 per 5% increase, 95% CI 1.45–2.02, *P* < 0.001) and peak troponin T (OR 0.85, 95% CI 0.81–0.90, *P* < 0.001) were independently associated with LVEF recovery.

**Table 2:** Predictors of LVEF recovery in patients with baseline reduced LVEF

	Univariate		Multivariate	
	OR (95% CI)	*P* value	OR (95% CI)	*P* value
Age, years	0.99 (0.98–1.01)	0.222		
Male	0.80 (0.51–1.25)	0.324		
Hypertension	0.99 (0.70–1.39)	0.935		
Diabetes	0.79 (0.56–1.11)	0.170		
Prior PCI	0.88 (0.30–2.57)	0.815		
Smoking	1.02 (0.72–1.43)	0.918		
Anterior MI	0.37 (0.25–0.53)	< 0.001	0.55 (0.36–0.84)	0.006
Killip class II–IV	0.72 (0.45–1.15)	0.164		
SBP (per 10 mmHg increase)	0.99 (0.92–1.05)	0.689		
Heart rate (per 10 bpm increase)	0.84 (0.77–0.92)	< 0.001	0.88 (0.79–0.98)	0.021
Out-of-hospital cardiac arrest	2.07 (0.80–5.34)	0.133		
Multivessel disease	1.11 (0.79–1.56)	0.549		
TIMI flow 0–1 before PCI	0.74 (0.51–1.06)	0.102		
Usage of IABP	0.69 (0.39–1.23)	0.210		
Baseline LVEF (per 5% increase)	1.88 (1.60–2.20)	< 0.001	1.71 (1.45–2.02)	< 0.001
Initial creatinine, umol/L	1.00 (0.99–1.00)	0.122		
Peak Troponin *T*, ng/mL	0.86 (0.82–0.90)	< 0.001	0.85 (0.81–0.90)	< 0.001
ACEIs/ARBs	1.22 (0.86–1.71)	0.264		
β-receptor blockers	0.59 (0.38–0.92)	0.020	0.79 (0.47–1.33)	0.378

*OR* odds ratio, *95% CI* 95% confidence interval, *LVEF* Left ventricular ejection fraction, *PCI* Percutaneous coronary intervention, *MI* myocardial infarction, *SBP* Systolic blood pressure, *TIMI* Thrombolysis in Myocardial Infarction, *IABP* Intra-aortic balloon pump, *ACEIs* Angiotensin-converting enzyme inhibitors, *ARBs* Angiotensin II receptor blockers

### LVEF trajectories and outcomes

**Fig. 3:**
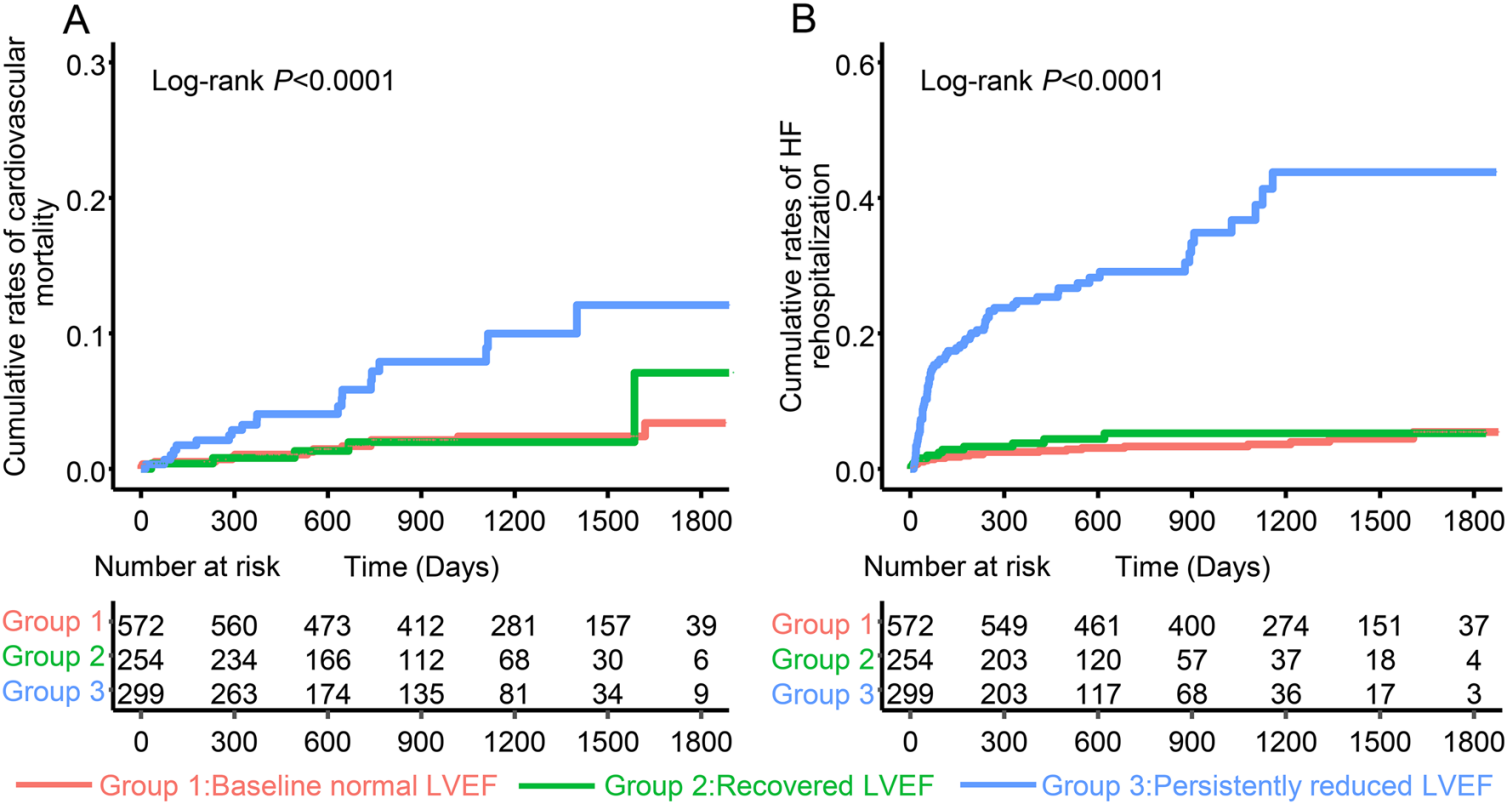
Kaplan–Meier curves for cardiovascular mortality (**A**) and HF rehospitalization (**B**) among baseline normal LVEF group, recovered LVEF group and persistently reduced LVEF group. *HF* heart failure, *LVEF* Left ventricular ejection fraction

During a median follow-up of 37.5(19.9–50.1) months, 38 cases of cardiovascular mortality occurred. Of which, 13 (2.3%) patients with baseline normal LVEF, 5 (2.0%) patients with recovered LVEF, and 20 (6.7%) patients with persistently reduced LVEF (Fig. 3A, Log-rank *P* < 0.001). When using the baseline normal LVEF group as reference, patients with recovered LVEF had no relationship with cardiovascular mortality (HR 1.10, 95% CI 0.39–3.08, *P* = 0.863), while patients with persistently reduced LVEF had an increased risk of cardiovascular mortality (HR 3.89, 95% CI 1.93–7.85, *P* < 0.001). The increased risk remained significant in patients with persistently reduced LVEF after fully adjusting for confounders (HR 7.49, 95% CI 1.94–28.87, *P* = 0.003). (Table 3).

**Table 3:** Univariate and multivariate hazard ratio for outcomes

	Univariate		Multivariate	
	HR (95% CI)	*P* value	HR (95% CI)	*P* value
Cardiovascular mortality				
Baseline normal LVEF	1.00	–	1.00	–
Recovered LVEF	1.10 (0.39–3.08)	0.863	1.95 (0.52–7.34)	0.325
Persistently Reduced LVEF	3.89 (1.93–7.85)	< 0.001	7.49 (1.94–28.87)	0.003
HF rehospitalization				
Baseline normal LVEF	1.00	–	1.00	–
Recovered LVEF	1.62 (0.80–3.30)	0.180	0.79 (0.34–1.86)	0.793
Persistently Reduced LVEF	9.66 (5.97–15.64)	< 0.001	3.54 (1.56–8.06)	0.003

Baseline normal LVEF was treated as reference. Multivariate adjusted for age, sex, hypertension, diabetes, chronic kidney disease, prior PCI, prior stroke, smoking, heart rate, SBP, Killip class II–IV, out-of-hospital cardiac arrest, peak troponin T, initial creatinine, multivessel disease, usage of IABP, TIMI flow 0–1 before PCI, baseline LVEF, ACEIs/ARBs at discharge, and β-blockers at discharge

*HR* hazards ratio, *95% CI* 95% confidence interval, *LVEF* Left ventricular ejection fraction, *HF* heart failure, *PCI* Percutaneous coronary intervention, *SBP* Systolic blood pressure, *IABP* Intra-aortic balloon pump, *TIMI* Thrombolysis in Myocardial Infarction, *ACEIs* Angiotensin-converting enzyme inhibitors, *ARBs* Angiotensin II receptor blockers

A similar pattern was observed for HF rehospitalization. During the follow-up, 111 cases of HF rehospitalization occurred. Among them, 22 (3.8%) patients in the baseline normal LVEF group, 12 (4.7%) patients in the recovered LVEF group, and 77 (25.8%) patients in the persistently reduced LVEF group (Fig. 3B, Log-rank *P* < 0.001). Compared to patients with baseline normal LVEF, the HR for HF rehospitalization was not significantly increased in patients with recovered LVEF (HR 1.62, 95% CI 0.80–3.30, *P* = 0.180), while the HR for HF rehospitalization was significantly increased in patients with persistently reduced LVEF (HR 9.66, 95% CI 5.97–15.64, *P* < 0.001). Patients with persistently reduced LVEF remained at an increased risk of HF rehospitalization after full adjustment (HR 3.54, 95% CI 1.56–8.06 *P* = 0.003) (Table 3).

### Sensitivity analysis

In this sensitivity analysis, we used a model with 3 latent classes to better elucidate our findings. As displayed in the Supplementary Fig. 2, patients were assigned into three trajectories based on the LCTM and they were labeled as greatly recovered LVEF, mildly recovered LVEF, and persistently reduced LVEF, respectively. After full adjustment, patients with persistently reduced LVEF had an increased risk of cardiovascular mortality (adjusted HR 7.93, 95% CI 1.53–41.79, *P* = 0.014) and HF rehospitalization (adjusted HR 7.05, 95% CI 2.79–17.80, *P* < 0.001) when utilizing baseline normal LVEF as a reference. While mildly recovered LVEF also identified patients at a heightened risk of cardiovascular mortality (fully adjusted HR 5.49, 95% CI 1.36–22.19, *P* = 0.017) compared with patients with baseline normal LVEF (Supplementary Fig. 3).

## Discussion

In this study, throughout a 3-year follow-up, we identified two distinct LVEF trajectories in 553 patients with a baseline reduced LVEF after STEMI, with 45.9% experiencing LVEF recovery and 54.1% suffering persistent LVEF reduction. We also demonstrated that lower peak troponin T, higher baseline LVEF, non-anterior MI and lower heart rates were independently associated with LVEF recovery. After adjusting for confounders, patients with persistently reduced LVEF had a higher risk of cardiovascular mortality and HF rehospitalization than patients with baseline normal LVEF, but patients with recovered LVEF had no such risk.

Few studies to date explored the long-term LVEF trajectories after MI. This study identified two distinct LVEF trajectories over a 3-year follow-up in 553 patients with a baseline reduced LVEF after STEMI by LCTM. 45.9% of patients experienced LVEF recovery, presenting with a gradual increase in LVEF during the first 9 months and reaching a plateau after that, and the rest of the patients suffered from persistent LVEF reduction at a lower level. Our result was consistent with the previous studies on LVEF recovery after MI. Wu et al. found that 42% of patients recovered their EF to ≥ 50% within 180 days post-MI in young patients aged ≤ 50 years [22]. A retrospective, single-center study of 554 STEMI patients with a baseline reduced LVEF (< 50%) reported that 54.0% of patients presented with complete recovery LVEF (≥ 50%) at 1-year follow-up[23]. Similar number was also observed in a recent study on LVEF recovery in patients with STEMI, with 47% of patients experiencing LVEF recovery after 3 months [16].

The change of LVEF post-MI is a dynamic process that depends on reversible myocardial stunning, irreversible necrosis and medication modification. Previous studies have shown that myocardial stunning is a phenomenon in which myocardial contractile function remains depressed but recovered within weeks after revascularization [10, 24]. Contrary, LV remodeling triggered by irreversible necrosis in the core of the infarct zone affected the long-term LVEF recovery [25, 26]. Besides, the current guidelines recommended that medication including angiotensin-converting enzyme inhibitors (ACEIs)/angiotensin II receptor blockers (ARBs), β-receptor blockers and mineralocorticoid receptor antagonist (MRA) can be used to improve myocardial remodeling after MI. However, most studies of LVEF recovery after MI report only a single LVEF reassessment, generally obtained at various time points (such as 3 months, 6 months or 1 year after MI), and the definition of LVEF recovery post-MI is unclear. Besides, no precise recommendations are provided regarding the timing of reassessment of LVEF in the current guidelines. Our study found that patients with recovered LVEF reached a plateau around 9 months after discharge. This suggests that reassessment of LVEF in the early phase after MI may misclassify patients who are in the stage of increasing LVEF but have relatively low LVEF at that point. Of note, in our study, patients with persistently reduced LVEF had a higher frequency of prescription of β-receptor blockers, which may be due to higher heart rates in these patients.

Predictors including lower peak troponin T, higher baseline LVEF, non-anterior MI and lower heart rates were associated with increased probability of LVEF recovery in our study. Consistent with prior evidence [16, 18, 27, 28], we found that patients with lower peak troponin T are more likely to experience LVEF recovery. Henning Steen et al. proved that higher peak cardiac troponin T represents a bigger infarct size in MI [29], which means more irreversible necrosis in the core of the infarct zone. Most studies also confirmed that higher baseline LVEF enhances the probability of recovery [16, 23], though some studies considered that patients with a lower baseline LVEF showed a more significant improvement in LV function [18, 30]. These differences may be due to participants' heterogeneity, inconsistent LVEF assessment methods, and non-uniform definitions of LVEF recovery. Our study also found that higher baseline LVEF was another predictor of LVEF recovery. Toronto et al. reported an inverse relationship between LVEF and infarct size determined by radionuclide measurement [31]. Based on this, we would like to speculate that patients with a higher baseline LVEF had smaller infarct size, a lower extent of myocardial ischemia, and a higher proportion of stunning myocardial in the infarct zone. So, these patients increased the possibility to experience LVEF recovery. Both LVEF and troponin T can reflect the extent of infarct size, but we still found they had different predictive values in our study because echocardiography could not distinguish myocardial stunning from myocardial necrosis. We thought that higher peak troponin T, instead of troponin T from other time points, reflected more irreversible myocardial necrosis in the core of the infarct zone, while higher baseline LVEF represented a higher proportion of reversible stunning myocardial. We also found that non-anterior MI was associated with LVEF recovery. Prior studies indicated that the involvement of the LMCA and/or LAD was a poor predictor of LVEF recovery [23] and the anterior location of MI was an independent predictor of LV remodeling [18]. This phenomenon can be explained by the greater magnitude of irreversible ischemic damage in anterior MI [32] and patients with anterior MI experienced more adverse LV remodeling than non-anterior MI patients [33, 34]. Though heart rates at admission and β-blockers at discharge showed significant statistical relation to LEVF recovery in univariate analysis, only heart rates at admission were related to LVEF recovery in the multivariate model. The DANAMI-3 trial determined that elevated heart rates are the independent correlate of infarct size and decreased LVEF [35]. The potential mechanisms of association between LV remodeling and elevated heart rates are as follows: higher heart rates lead to more oxygen demand, shorter coronary diastolic perfusion time, and indicate enhanced sympathetic excitation, thus exacerbating myocardial ischemia and hypoxia, finally promoting LV remodeling after MI [35–37].

It is well accepted that depressed LVEF is a predictor of poor prognosis in MI patients over the past decades [4, 38]. Furthermore, more and more data prove that LVEF recovery is associated with a better prognosis. A prospective study that enrolled 228 patients with AMI and LV dysfunction determined that patients who recovered their LVEF had lower rates of 5-year cardiac mortality and HF rehospitalization [9]. A real-world study confirmed that patients with no LVEF recovery (△LVEF ≤ 0%) had a higher risk of death compared with a modest (0% < △LVEF ≤ 10%) or large recovery (△LVEF > 10%) in LVEF[18]. Oscar et al. found that patients with LVEF recovery (LVEF ≥ 50%) 1-year post-MI decreased their risk of all-cause and cardiovascular mortality compared with patients who did not [23]. Similar results were also observed in patients who experienced their first MI at a young age (< 50 years)[22]. In addition, Jeroen Dauw et al. reported that patients with recovered LVEF shared an equal risk of all-cause mortality and HF hospitalization compared with patients with baseline normal LVEF; on the other hand, patients with persistent LVEF reduction remained at higher risk [16]. So far, few studies on the association between long-term LVEF trajectories after STEMI and prognosis have been reported. Interestingly, our study indicated that patients showing persistently reduced LVEF trajectory had a higher risk of cardiovascular mortality and HF rehospitalization than patients with baseline normal LVEF. In contrast, patients presenting with recovered LVEF trajectory reduced their risk of cardiovascular mortality and HF rehospitalization to the level of patients with baseline normal LVEF. The current guidelines provided no exact recommendations for reevaluation of LVEF in MI after discharge. Our findings implied that reevaluating LVEF after discharge in STEMI can provide information on risk stratification and is a benefit of early recognizing the need to apply anti-remodeling therapies, including ACEIs, ARBs, ARNIs, β-blockers, or MRA.

This study should be interpreted carefully because of some limitations. First, this is a retrospective, observational, single-center research, though the sample size is considerable. Second, our LCTM only identified two trajectories (recovered LVEF and persistently reduced LVEF) based on minimum BIC and the highest mean posterior probability. Although the sensitivity analysis using a model with three latent classes revealed three different trajectories (greatly recovered LVEF, mildly recovered LVEF and persistently reduced LVEF), and patients with persistently reduced LVEF had worse outcomes, which was consistent with the primary analysis. The other possible trajectories (like a trajectory that LVEF first rises and then reduces, or a trajectory that LVEF first reduces first then rises) still exists in the real world. The following are two possible explanations: on the one hand, we believe that recovered (greatly or mildly) and persistently reduced trajectories are predominant in clinical practice, on the other hand, many follow-up LVEF values were missing at each time point due to the observational design of the present study. This indicates that a study with bigger sample size, prospective design, and time-scheduled LVEF measurements should be conducted in the future. Fourth, we did not analyze the LVEF in patients with baseline normal LVEF, though a previous study showed most of these patients remained normal LVEF at follow-up [39]. Finally, some patients with baseline reduced LVEF were excluded due to inaccessible LVEF after discharge. Despite the baseline characteristics of these patients were comparable to those of patients included, the selection bias may still exist.

## Conclusion

In summary, two distinct LVEF trajectories over a 3-year follow-up were identified. Patients with persistently reduced LVEF had a higher risk of cardiovascular mortality and HF rehospitalization than those with baseline normal LVEF, while the association was not pronounced in patients with recovered LVEF. In addition, several baseline characteristics, including lower peak troponin T, higher baseline LVEF, non-anterior MI and lower heart rates, can be used to predict long-term LVEF recovery. These findings suggest that repeated LVEF evaluation after discharge can help stratification in STEMI patients, especially patients with a higher risk of persistent LVEF reduction.

## Supplementary Information

Below is the link to the electronic supplementary material.

Supplementary file1 (DOCX 375 kb)

## References

[1] Mozaffarian D, Benjamin EJ, Go AS (2016). Heart disease and stroke statistics-2016 update: a report from the american heart association. Circulation.

[2] Mortensen MB, Falk E (2018). Primary prevention with statins in the elderly. J Am Coll Cardiol.

[3] Halkin A, Stone GW, Dixon SR (2005). Impact and determinants of left ventricular function in patients undergoing primary percutaneous coronary intervention in acute myocardial infarction. Am J Cardiol.

[4] Multicenter Postinfarction Research Group (1983). Risk stratification and survival after myocardial infarction. N Engl J Med.

[5] Lewis EF, Moye LA, Rouleau JL (2003). Predictors of late development of heart failure in stable survivors of myocardial infarction. J Am Coll Cardiol.

[6] Lewis EF, Velazquez EJ, Solomon SD (2008). Predictors of the first heart failure hospitalization in patients who are stable survivors of myocardial infarction complicated by pulmonary congestion and/or left ventricular dysfunction: a VALIANT study. Eur Heart J.

[7] Moller JE, Hillis GS, Oh JK (2006). Wall motion score index and ejection fraction for risk stratification after acute myocardial infarction. Am Heart J.

[8] Ottervanger JP, van’t Hof AW, Reiffers S (2001). Long-term recovery of left ventricular function after primary angioplasty for acute myocardial infarction. Eur Heart J.

[9] Parodi G, Memisha G, Carrabba N (2007). Prevalence, predictors, time course, and long-term clinical implications of left ventricular functional recovery after mechanical reperfusion for acute myocardial infarction. Am J Cardiol.

[10] Kloner RA (2020). Stunned and hibernating myocardium: where are we nearly 4 Decades later?. J Am Heart Assoc.

[11] Rodriguez-Palomares JF, Gavara J, Ferreira-Gonzalez I (2019). Prognostic value of initial left ventricular remodeling in patients with reperfused STEMI. JACC Cardiovasc Imaging.

[12] van der Bijl P, Abou R, Goedemans L (2020). Left ventricular post-infarct remodeling: implications for systolic function improvement and outcomes in the modern era. JACC Heart Fail.

[13] Ponikowski P, Voors AA, Anker SD (2016). 2016 ESC Guidelines for the diagnosis and treatment of acute and chronic heart failure: the Task Force for the diagnosis and treatment of acute and chronic heart failure of the European Society of Cardiology (ESC)Developed with the special contribution of the Heart Failure Association (HFA) of the ESC. Eur Heart J.

[14] Sjöblom J, Muhrbeck J, Witt N (2014). Evolution of left ventricular ejection fraction after acute myocardial infarction: implications for implantable cardioverter-defibrillator eligibility. Circulation.

[15] Otero-García O, Cid-Álvarez AB, Juskova M (2021). Prognostic impact of left ventricular ejection fraction recovery in patients with ST-segment elevation myocardial infarction undergoing primary percutaneous coronary intervention: analysis of an 11-year all-comers registry. Eur Heart J Acute Cardiovasc Care.

[16] Dauw J, Martens P, Deferm S (2021). Left ventricular function recovery after ST-elevation myocardial infarction: correlates and outcomes. Clin Res Cardiol.

[17] Chew DS, Heikki H, Schmidt G (2018). Change in left ventricular ejection fraction following first myocardial infarction and outcome. JACC Clin Electrophysiol.

[18] Chew DS, Wilton SB, Kavanagh K (2018). Left ventricular ejection fraction reassessment post-myocardial infarction: current clinical practice and determinants of adverse remodeling. Am Heart J.

[19] Nagin DS, Tremblay RE (2001). Analyzing developmental trajectories of distinct but related behaviors: a group-based method. Psychol Methods.

[20] Proust-Lima C, Letenneur L, Jacqmin-Gadda H (2007). A nonlinear latent class model for joint analysis of multivariate longitudinal data and a binary outcome. Stat Med.

[21] Cleveland WS, Devlin SJ (1988). Locally weighted regression: an approach to regression analysis by local fitting. J Am Stat Assoc.

[22] Wu WY, Biery DW, Singh A (2020). Recovery of left ventricular systolic function and clinical outcomes in young adults with myocardial infarction. J Am Coll Cardiol.

[23] Otero-Garcia O, Cid-Alvarez AB, Juskova M, et al. (2021) Prognostic impact of left ventricular ejection fraction recovery in patients with ST-segment elevation myocardial infarction undergoing primary percutaneous coronary intervention: analysis of an 11-year all-comers registry. Eur Heart J Acute Cardiovasc Care 10.1093/ehjacc/zuab058 34327531

[24] Hoole SP, Heck PM, White PA (2010). Stunning and cumulative left ventricular dysfunction occurs late after coronary balloon occlusion in humans insights from simultaneous coronary and left ventricular hemodynamic assessment. JACC Cardiovasc Interv.

[25] Heusch G, Libby P, Gersh B (2014). Cardiovascular remodelling in coronary artery disease and heart failure. Lancet (Lond Engl).

[26] Camici PG, Prasad SK, Rimoldi OE (2008). Stunning, hibernation, and assessment of myocardial viability. Circulation.

[27] Hallen J, Jensen JK, Fagerland MW (2010). Cardiac troponin I for the prediction of functional recovery and left ventricular remodelling following primary percutaneous coronary intervention for ST-elevation myocardial infarction. Heart (Br Cardiac Soc).

[28] Brooks GC, Lee BK, Rao R (2016). Predicting persistent left ventricular dysfunction following myocardial infarction: the PREDICTS study. J Am Coll Cardiol.

[29] Steen H, Giannitsis E, Futterer S (2006). Cardiac troponin T at 96 h after acute myocardial infarction correlates with infarct size and cardiac function. J Am Coll Cardiol.

[30] Ndrepepa G, Mehilli J, Martinoff S (2007). Evolution of left ventricular ejection fraction and its relationship to infarct size after acute myocardial infarction. J Am Coll Cardiol.

[31] Burns RJ, Gibbons RJ, Yi Q (2002). The relationships of left ventricular ejection fraction, end-systolic volume index and infarct size to six-month mortality after hospital discharge following myocardial infarction treated by thrombolysis. J Am Coll Cardiol.

[32] Masci PG, Ganame J, Francone M (2011). Relationship between location and size of myocardial infarction and their reciprocal influences on post-infarction left ventricular remodelling. Eur Heart J.

[33] Bourke S, Conroy RM, Mulcahy R (1988). Aetiological and prognostic correlates of site of myocardial infarction. Eur Heart J.

[34] Hands ME, Lloyd BL, Robinson JS (1986). Prognostic significance of electrocardiographic site of infarction after correction for enzymatic size of infarction. Circulation.

[35] Inoue T, Iseki K, Ohya Y (2013). Heart rate as a possible therapeutic guide for the prevention of cardiovascular disease. Hypertens Res Off J Jpn Soc Hyperten.

[36] French BA, Kramer CM (2007). Mechanisms of post-infarct left ventricular remodeling. Drug Discov Today Dis Mech.

[37] Colin P, Ghaleh B, Monnet X (2004). Effect of graded heart rate reduction with ivabradine on myocardial oxygen consumption and diastolic time in exercising dogs. J Pharmacol Exp Ther.

[38] Rouleau JL, Talajic M, Sussex B, Potvin L, Warnica W, Davies RF, Gardner M, Stewart D, Plante S, Dupuis R (1996). Myocardial infarction patients in the 1990s–their risk factors, stratification and survival in Canada: the Canadian Assessment of Myocardial Infarction (CAMI) Study. J Am Coll Cardiol.

[39] Soholm H, Lonborg J, Andersen MJ, Vejlstrup N, Engstrom T, Moller JE, Hassager C (2015). Repeated echocardiography after first ever ST-segment elevation myocardial infarction treated with primary percutaneous coronary intervention–is it necessary?. Eur Heart J Acute Cardiovasc Care.

