# Supplementary material for: Predictors and prognostic impact of left ventricular ejection fraction trajectories in patients with ST-segment elevation myocardial infarction
Source: Aging Clin Exp Res. 2022 Feb 11;34(6):1429–38. doi: 10.1007/s40520-022-02087-y (PMC9151544; doi:10.1007/s40520-022-02087-y)
Supplement: Supplementary file 1 — Supplementary file1 (DOCX 375 kb) [file 40520_2022_2087_MOESM1_ESM.docx]

Supplementary material

Supplementary Table1. Parameter estimates for the latent class trajectory model.

Supplementary Table 2. Demographic, clinical, and therapeutic characteristics of baseline reduced LVEF patients with and without follow-up LVEF.

Supplementary Figure 1. Distribution of the number of LVEF measurements performed per patient.

Supplementary Figure 2. Comparison of long-term LVEF trajectories among patients with greatly recovered LVEF (blue), mildly recovered LVEF (green), and persistently reduced LVEF (yellow) in sensitivity analysis. *P*=0.751 for LVEF trajectory changes for patients with persistently reduced LVEF, *P*<0.001 for LVEF trajectory changes for patients with mildly recovered LVEF; *P*<0.001 for LVEF trajectory changes for patients with greatly recovered LVEF; *P*<0.001 for comparison among the three groups. Shaded regions represent 95% confidence interval. The table shows the number of LVEF values at each time points.

Supplementary Figure 3. Adjusted HR and 95% CI for outcomes in sensitivity analysis.

Baseline normal LVEF was treated as reference. Multivariate adjusted for age, sex, hypertension, diabetes, chronic kidney disease, prior PCI, prior stroke, smoking, heart rate, SBP, Killip class II~IV, out-of-hospital cardiac arrest, peak troponin T, initial creatinine, multivessel disease, usage of IABP, TIMI flow 0~1 before PCI, baseline LVEF, ACEIs/ARBs at discharge, and β-blockers at discharge.

HR: hazards ratio; 95% CI: 95% confidence interval; LVEF: Left ventricular ejection fraction; HF: heart failure; PCI: Percutaneous coronary intervention; SBP: Systolic blood pressure; IABP: Intra-aortic balloon pump; TIMI: Thrombolysis in Myocardial Infarction; ACEIs: Angiotensin-converting enzyme inhibitors; ARBs: Angiotensin II receptor blockers.

| Supplementary Table 1. Parameter estimates for the latent class trajectory model. | | | | | |
| --- | --- | --- | --- | --- | --- |
| Number of latent classes | Polynomial degree | BIC | Percentage patients  per class (%) | Mean posterior  probabilities | Posterior probabilities >0.7 (%) |
| 2 | second-order | 11103.98 | 54.1/45.9 | 0.87/0.84 | 80.6/82.3 |
| 3 | second-order | 11125.92 | 35.1/23.9/41.0 | 0.81/0.63/0.80 | 72.7/31.1/71.4 |
| 4 | second-order | 11150.37 | 40.0/7.1/10.1/42.8 | 0.78/0.55/0.53/0.74 | 68.8/25.6/12.5/59.1 |
| 5 | second-order | 11181.58 | 40.7/14.4/8.0/19.9/17.0 | 0.76/0.49/0.51/0.42/0.56 | 65.7/15.0/18.2/0.0/19.1 |

BIC: Bayesian Information Criterion.

| Supplementary Table 2. Demographic, clinical, and therapeutic characteristics of baseline reduced LVEF patients with and without follow-up LVEF. | | | |
| --- | --- | --- | --- |
|  | With follow-up LVEF N=553 | Without follow-up LVEF N=54 | *P* value |
| Age, years | 64.3±11.3 | 31.3±10.4 | 0.969 |
| Male | 458(82.8%) | 48(88.9%) | 0.253 |
| BMI, Kg/m^2^ | 24.3(22.8-26.3) | 24.2(22.9-25.6) | 0.395 |
| Hypertension | 345(62.4%) | 35(64.8%) | 0.725 |
| Diabetes | 213(38.5%) | 16(29.6%) | 0.198 |
| Chronic kidney disease | 27(4.9%) | 3(5.6%) | 1.000 |
| Prior stroke | 41(7.4%) | 5(9.3%) | 0.826 |
| Prior PCI | 14(2.5%) | 1(1.9%) | 1.000 |
| Smoking | 334(60.4%) | 32(59.3%) | 0.870 |
| Anterior MI | 374(67.6%) | 33(61.1%) | 0.331 |
| Killip class II~IV | 87(15.7%) | 12(22.2%) | 0.218 |
| SBP, mmHg | 136.8±25.4 | 134.5±26.7 | 0.528 |
| Heart rate, beats/min | 83.0±19.5 | 82.0±17.7 | 0.708 |
| Out-of-hospital cardiac arrest | 19(3.4%) | 3(5.6%) | 0.679 |
| Multivessel disease | 325(58.9%) | 33(61.1%) | 0.750 |
| TIMI flow 0~1 before PCI | 385(69.6%) | 37(68.5%) | 0.867 |
| Usage of IABP | 53(9.6%) | 3(5.6%) | 0.465 |
| Baseline LVEF, % | 40.0(36.0-45.0) | 40.0(34.0-45.0) | 0.501 |
| Initial creatinine, umol/L | 78.9(67.3-93.2) | 83.8(72.2-91.2) | 0.415 |
| Peak troponin T, ng/mL | 8.09(3.68-10.00) | 8.15(3.08-10.00) | 0.720 |
| Medication at discharge |  |  |  |
| Aspirin | 532(96.2%) | 51(94.4%) | 0.789 |
| Anti-P2Y12 receptors | 546(98.7%) | 54(100.0%) | 0.870 |
| Statins | 537(97.1%) | 52(96.3%) | 1.000 |
| ACEIs/ARBs | 330(59.7%) | 36(66.7%) | 0.316 |
| β-receptor blockers | 460(83.2%) | 46(85.2%) | 0.706 |

LVEF: Left ventricular ejection fraction; BMI: Body mass index; PCI: Percutaneous coronary intervention; MI: myocardial infarction; SBP: Systolic blood pressure; TIMI: Thrombolysis in Myocardial Infarction; IABP: Intra-aortic balloon pump; ACEIs: Angiotensin-converting enzyme inhibitors; ARBs: Angiotensin II receptor blockers.


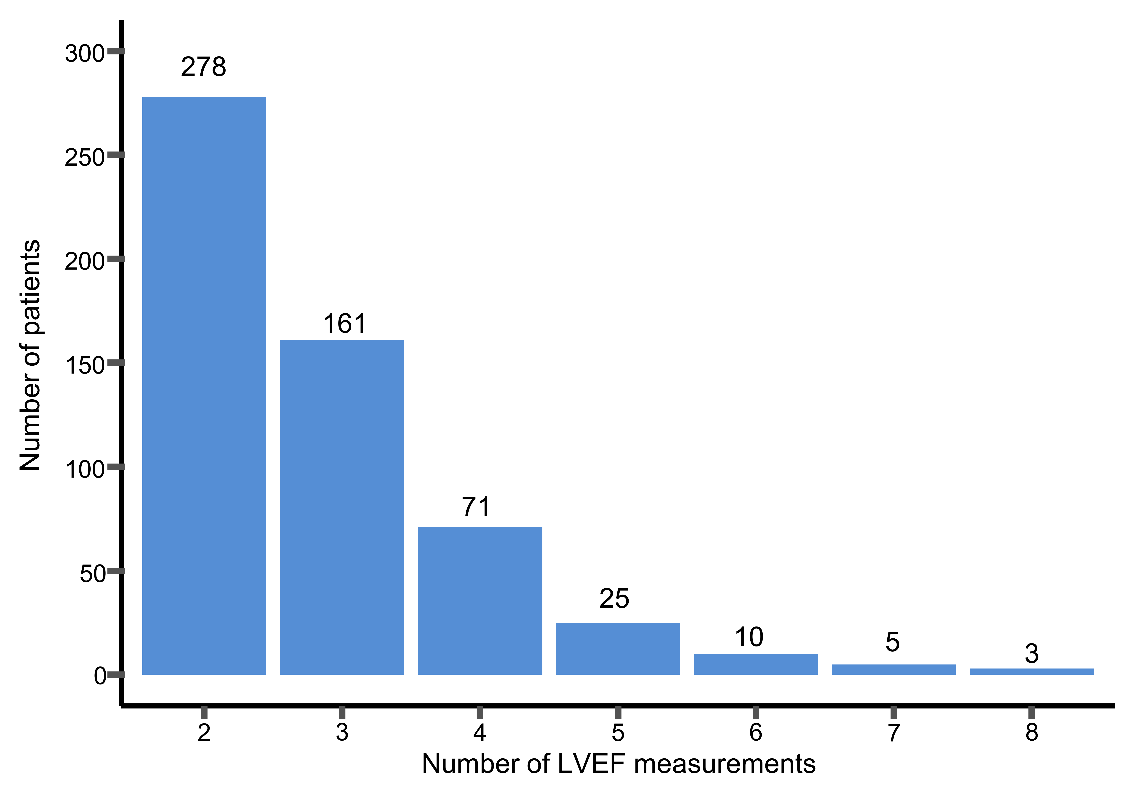


Supplementary Figure 1. Distribution of the number of LVEF measurements performed per patient.


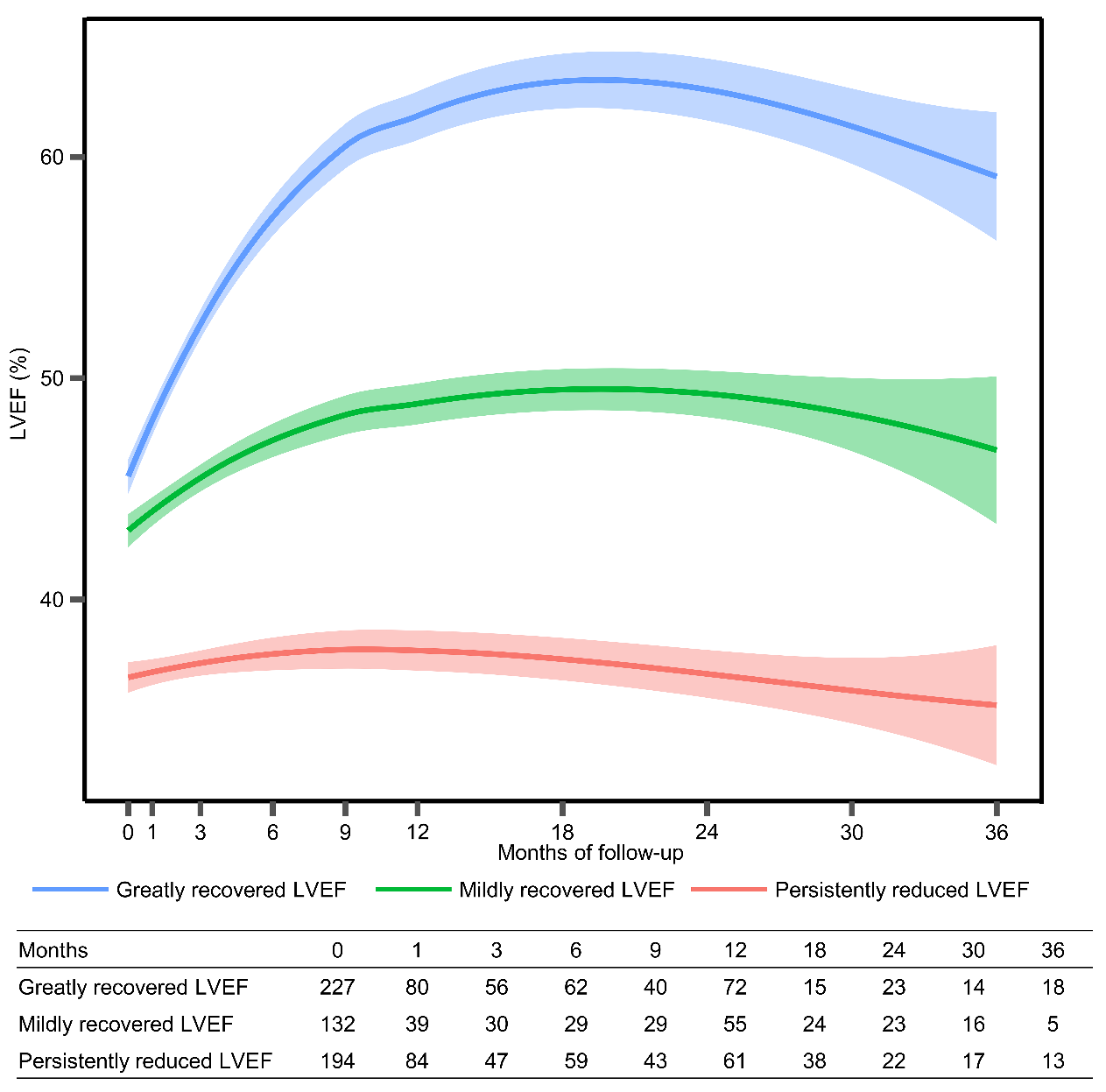


Supplementary Figure **2**: Comparison of long-term LVEF trajectories among patients with greatly recovered LVEF (blue), mildly recovered LVEF (green), and persistently reduced LVEF (orange) in sensitivity analysis. *P*=0.751 for LVEF trajectory changes for patients with persistently reduced LVEF, *P*<0.001 for LVEF trajectory changes for patients with mildly recovered LVEF; *P*<0.001 for LVEF trajectory changes for patients with greatly recovered LVEF; *P*<0.001 for comparison among the three groups. Shaded regions represent 95% confidence interval. The table shows the number of LVEF values at each time points.


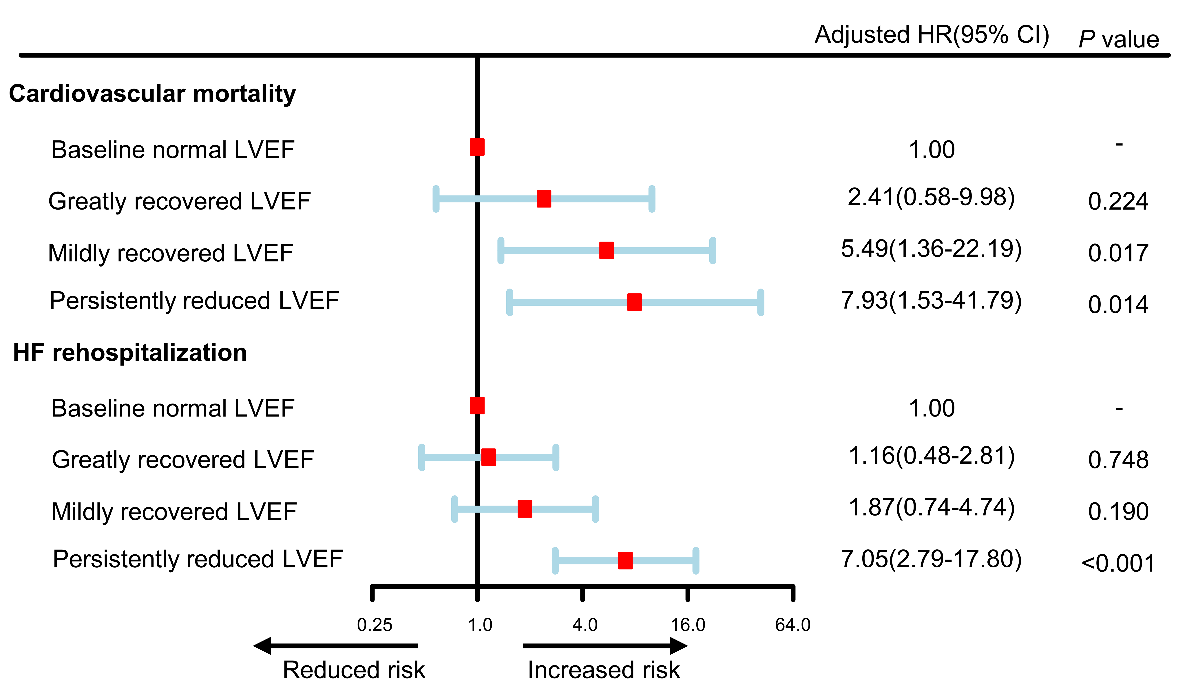


Supplementary Figure **3**: Adjusted HR and 95% CI for outcomes in sensitivity analysis.

Baseline normal LVEF was treated as reference. Multivariate adjusted for age, sex, hypertension, diabetes, chronic kidney disease, prior PCI, prior stroke, smoking, heart rate, SBP, Killip class II~IV, out-of-hospital cardiac arrest, peak troponin T, initial creatinine, multivessel disease, usage of IABP, TIMI flow 0~1 before PCI, baseline LVEF, ACEIs/ARBs at discharge, and β-blockers at discharge.

HR: hazards ratio; 95% CI: 95% confidence interval; LVEF: Left ventricular ejection fraction; HF: heart failure; PCI: Percutaneous coronary intervention; SBP: Systolic blood pressure; IABP: Intra-aortic balloon pump; TIMI: Thrombolysis in Myocardial Infarction; ACEIs: Angiotensin-converting enzyme inhibitors; ARBs: Angiotensin II receptor blockers.
